# Dynamic impacts of public health events on price fluctuations in broiler industry Chain in China: Evidence from COVID-19 epidemic

**DOI:** 10.1371/journal.pone.0307490

**Published:** 2024-07-22

**Authors:** Ning Xie, Haixin Fan, Xiaochun Liu, Feng Ye, Zhenlin Weng

**Affiliations:** 1 School of Economics and Management, Jiangxi Agricultural University, Nanchang, Jiangxi, P.R. China; 2 Jiangxi Rural Revitalization Strategy Research Institute, Jiangxi Agricultural University, Nanchang, Jiangxi, P.R. China; University of Agriculture Faisalabad, PAKISTAN

## Abstract

Using weekly data from January 2020 to December 2021 on the prices of various links in the Chinese broiler industry chain and the COVID-19 epidemic, we employed a time-varying parametric vector auto-regressive (TVP-VAR) model to investigate the dynamic effects of public health events on price fluctuations of upstream, midstream, and downstream products in the Chinese broiler industry chain. Our findings showed that the COVID-19 epidemic had different effects on the prices of various broiler products, both in direction and magnitude, at different lags and time intervals. Chicken and live chicken prices were impacted the most, followed by broiler chick prices, while broiler feed prices were impacted the least. The epidemic constantly impacted broiler chick and chicken prices, while its effect on live chicken prices was initially negative but turned positive afterwards. Additionally, the impact of the COVID-19 epidemic on broiler product prices consistently increased with more extended lag periods. The impulse responses at different epidemic time points were heterogeneous. With the results of this study, policy recommendations can be suggested to relevant government departments to optimize the prevention and control measures for public health emergencies and ensure price stability in the broiler industry.

## Introduction

Chicken is an economical and essential source of animal protein, making it an indispensable part of the daily diet for many citizens in China [1, 2]. The development of the broiler industry also contributes to improving the overall nutritional intake, optimizing the food structure, and enhancing food security [3]. Since its establishment in the 1980s, China’s broiler industry has undergone rapid development, making it the world’s second-largest producer and consumer of chicken meat [4]. In 2021, the broiler slaughter volume in China reached 11.83 billion, indicating a 7.4% increase from the previous year. Similarly, broiler consumption in China reached 20.904 million tons, a 6.7% increase from the previous year. Therefore, stabilizing broiler prices not only protects the standard of living of the population, but also promotes the sustainable development of the broiler industry [5, 6].

However, China’s broiler breeding industry has had frequent price fluctuations in recent years. The factors affecting this fluctuation include breeding costs, transportation costs, substitute prices, residents’ income levels and consumption preferences, financial factors, and unexpected events [7–9]. Among these factors, sudden major public events significantly impact price fluctuations in the broiler industry chain [10, 11]. In particular, after the COVID-19 outbreak in 2020, broiler prices fluctuated dramatically in a short period of time, which had a significant impact on residential chicken consumption. In this paper, we analyzed the impact of COVID-19 Epidemic on broiler price volatility using broiler price data at the national average level in China. Our findings can provide policy guidance for the broiler industry in the face of other public health event occurrences in the future.

The existing literature has done a lot of exploration around the impact of animal diseases on livestock and poultry prices. Outbreaks of animal diseases had a tremendous adverse effect on the entire poultry industry chain, resulting in substantial economic losses [12–14]. Saghaian et al. [15] and Cai and Tao [16] discovered that the avian influenza epidemic in 2005 hurt poultry product prices, and the extent of price adjustment depended on the type of poultry product. Hassouneh et al. [17] created a Food Scare Information Index (FSII) to examine its influence on poultry market prices in Egypt. Likewise, Mutlu et al. [18] utilized the Food Scare Information Index and investigated how avian flu outbreaks affected prices in the Turkish poultry market. Their findings revealed that retail prices rapidly deviated from long-term equilibrium after a price shock, while producer prices were slowly responsive due to price stickiness. Several scholars have also researched the indirect effects of African swine fever on the poultry market and found that although this disease primarily impacted the price of pork, it also caused some price fluctuations in chicken [19, 20].

There is also a part of the literature that focuses on the impact of COVID-19 on agricultural price inflation. The COVID-19 epidemic’s outbreak has tremendously affected the Chinese and global economies [21–25]. After COVID-19, agricultural prices in China continued to rise [26–28]. The COVID-19 outbreak triggered a severe shortage of broiler feed, leading to significant disruptions in product replenishment, sales, and slaughter, as well as fluctuations in broiler product consumption, thus had a significant impact on the stability of the broiler industry chain [29]. Compared to animal epidemics like avian influenza and African swine fever, the influence mechanism of public events like COVID-19 on the broiler industry’s market prices is vastly distinct. Animal epidemics affect broiler prices by targeting supply and consumer behavior [30], whereas the impact of COVID-19 on the broiler industry relies on prevention and control [31, 32]. Currently, most studies regarding the impact of COVID-19 on the broiler industry focus on qualitative analysis [33–35], with fewer conducting quantitative analyses. Zhong and Xu [36] constructed a VAR model and observed that the impact of the COVID-19 epidemic shock on live chicken and chicken prices lasted approximately six months.

However, existing study did not account for potential structural mutations or analyze the evolving impact on the industry. Given that the COVID-19 epidemic has persisted in China for three years with constantly changing policies, a TVP-VAR model can accurately capture the time-varying interaction relationships among relevant variables from a dynamic perspective [37, 38]. Therefore, the objective of this study is to use TVP-VAR model to analyze the impact of the COVID-19 pandemic on the market prices of the broiler industry chain in China. TVP-VAR model improves the adaptability and flexibility of the model by allowing the model parameters to vary over time, which enables it to more accurately capture the dynamic characteristics and structural changes in the data, produce more accurate estimates, and provide a powerful tool for economic analysis and policy formulation.

We attempt to make the following 3 contributions to the literature: (1) Developing a theoretical model based on the supply and demand shocks of the COVID-19 pandemic to showcase the price transmission channels of the broiler industry chain in China; (2) Implementing the TVP-VAR model to ascertain the time-varying features of the COVID-19 shocks on the market price fluctuations of the broiler industry chain in China; (3) Analyzing the different consequences of public health emergencies in compliance with various policies.

The remainder of this paper is organized as follows. Theoretical framework section illustrates the changes in supply and demand of the broiler market in response to the COVID-19 epidemic shock. Materials and methods section provides details of the data sources and research design. Results and discussion section builds up a TVP-VAR model to empirically test the impacts of the COVID-19 epidemic on the market price of broiler industry chain. Conclusions and policy recommendations section presents the conclusions and policy recommendations.

## Theoretical framework

**Fig 1:**
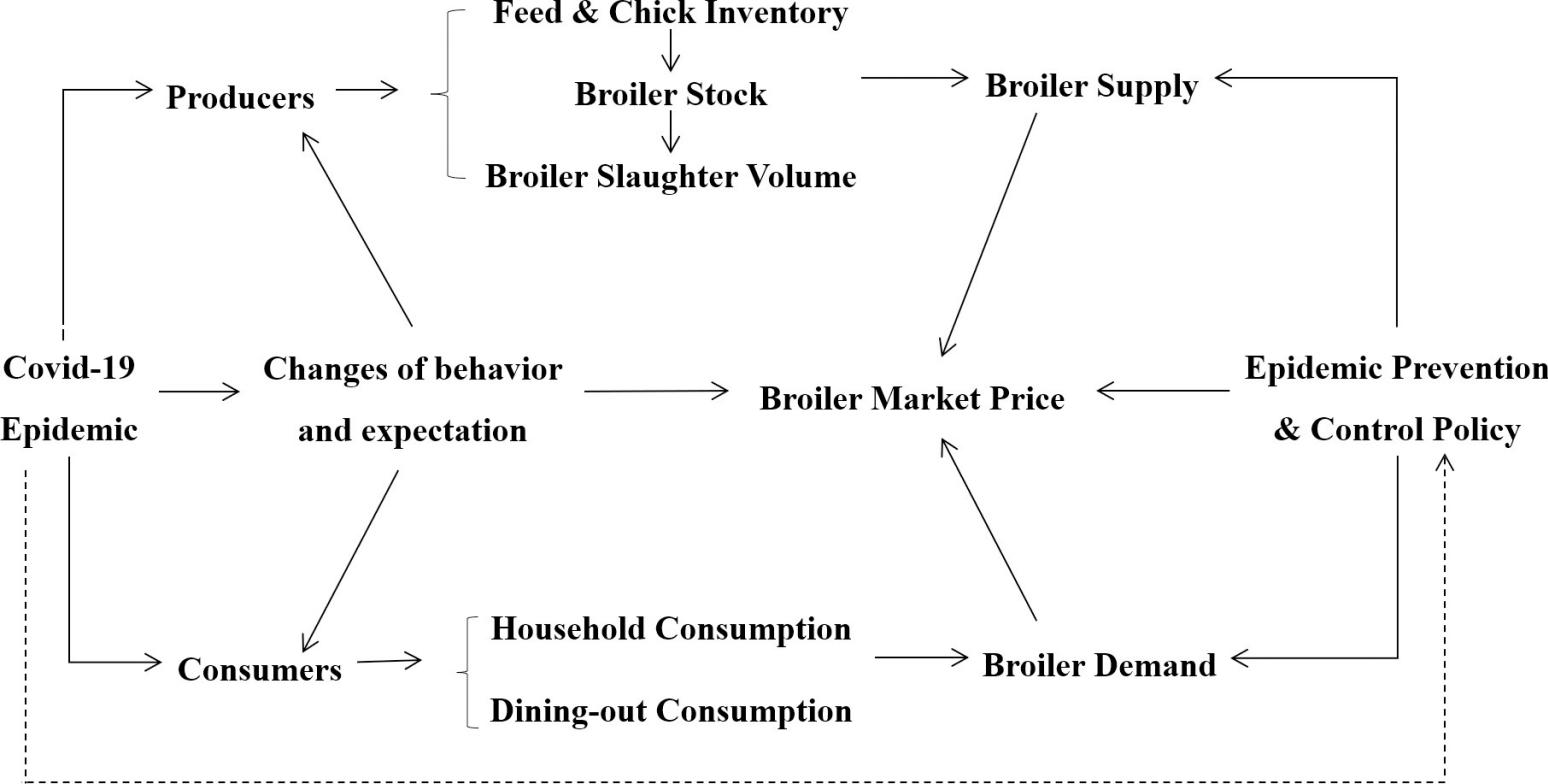
Transmission channels of COVID-19 epidemic on the price fluctuation of broiler industry chain.

According to the theory of supply and demand, the equilibrium price of broiler products is determined by the balance between market supply and demand. If the demand exceeds the supply, there will be an oversupply phenomenon leading to an increase in the equilibrium price of broiler products. Conversely, when the supply exceeds the demand, there will be a surplus of broiler products in the market, leading to a decrease in the equilibrium price. During public health emergencies like the COVID-19 epidemic, market price fluctuations in the broiler industry chain are also influenced by different stages of epidemic prevention and control policies. The COVID-19 epidemic affects the broiler market through three channels: producers, consumers, and policymakers, as shown in Fig 1.

In the broiler market, producers and consumers with varying behaviors and preferences exist. As the epidemic situation fluctuates in different periods, their behaviors and expectations alter accordingly. As a result, this transfers the epidemic shock effect to broiler production and consumption through supply and demand, and in turn, propelling shifts in broiler prices [38]. Unlike producers and consumers, policymakers’ actions and expectations are not solely influenced by changes in the broiler market. They consider the epidemic situation more comprehensively to develop appropriate policies for regulation and control. Assuming that the supply function of broiler products is *y*^*s*^ = a+δp, and the demand function of broiler products is *y*^*d*^ = −*b*+λp, where δ, λ, a, b are constants and greater than 0. p denotes broiler prices. *s* and *d* denote aggregate supply and demand.

The functional relationship among the changes in aggregate supply, aggregate demand, and price of the broiler market, after accounting for the impact of public health emergencies, can be summarized as follows.


$${\Delta y}^{s}=\alpha \Delta p+\varepsilon^{s},\;\alpha \geq 0$$ (1)

$$\Delta y^{d}=-\beta \Delta p+\varepsilon^{d},\;\beta \geq 0$$ (2)

The first equation represents the overall function for supply change, and the second equation represents the function for demand change in the broiler market. As a result of the COVID-19 outbreak, the broiler market has been impacted by simultaneous shocks in both supply and demand. Changes in broiler stock, slaughter, and consumer preferences have contributed to fluctuations in the equilibrium price of broiler products. In the short term, the equilibrium condition tends to be *y*^*s*^ = *y*^*d*^. Coefficients α and *β* denote the alterations in aggregate supply and demand attributed to pricing factors, while ε^s^ and *ε*^*d*^ illustrate the changes in supply and demand arising from the COVID-19 epidemic shock.

From the supply side, labor-intensive industries, such as broiler slaughter and processing plants, were severely affected by the COVID-19 outbreak. The wet, cold, and crowded working conditions in these plants made it easy for the virus to spread rapidly. In some plants, up to 70% of employees were infected with COVID-19, and almost half of the broiler slaughter and processing plants with outbreaks had to shut down [39]. As a result, millions of broilers had to be euthanized, leading to a decline in broiler stocking and slaughter and, ultimately, a decrease in broiler supply [40, 41]. Additionally, disseminating information regarding the COVID-19 outbreak would impact producers’ production decisions. Due to the volatile nature of public health crises, some broiler producers, tiny and medium-sized enterprises, and farmers might make "limited rational decisions" to reduce their breeding scale or exit the market by carefully considering potential risks and their capacity to bear them. This action further reduced broiler chickens’ supply and subsequently led to a significant impact on broiler prices.

From the demand side, dining out expenses accounted for much broiler consumption before the COVID-19 epidemic [42]. However, the outbreak of the COVID-19 epidemic led to the enforcement of strict home quarantine policies, resulting in the closure of various businesses, schools, and food and beverage establishments. Consequently, outdoor and group broiler consumption demand declined steadily [31]. Maples et al. [29] discovered a substantial decrease in the demand for outdoor poultry consumption. Subsequently, after implementing several favorable governmental policies, COVID-19 was effectively controlled, leading to the resumption of work, production, and school activities. As a result, the restaurant industry gradually recovered, and consumer confidence was restored. Additionally, with the proliferation of favorable media campaigns and increased consumer awareness of the health benefits of chicken consumption, there had been a surge in demand for chicken during the later period of the COVID-19 epidemic.

From the policy level, after the outbreak of the COVID-19 epidemic, the government introduced a series of policies to prevent its spread. As the situation evolved, these epidemic prevention and control policies were adjusted accordingly from 2020 to 2023. As mentioned earlier, these policy adjustments affected the supply and demand of broiler products. For example, in the beginning, strict home quarantine and logistics blockade policies were implemented in areas with severe outbreaks. These policies resulted in the closure of the catering industry and a notable reduction in the overall demand for broilers, labor shortages, and blockages in production and marketing. As a result, in production areas, broilers that had reached their intended slaughter period could not be marketed promptly, resulting in a surplus of live chicken supply and a significant decline in prices. Conversely, in sales areas, the supply was limited, leading to a noteworthy escalation in prices [32, 43]. Subsequently, in collaboration with provincial agricultural and rural departments, the Ministry of Agriculture and Rural Affairs put forth a range of support policies to secure the availability of crucial agricultural supplies and products, sustain the proper functioning of animal farming, and facilitate economic revival. As epidemic prevention and control measures gradually eased, the consumption of broiler poultry swiftly bounced back. Nevertheless, broiler production capacity had to be gradually restored, thus the supply and demand gap would persist until it normalized.

## Materials and methods

### Data

This study utilized weekly price data of broiler feed, broiler chicks, live chickens, and chicken in China from week 4 of 2020 to week 52 of 2021. We used average price data from China for our empirical analysis. This data was collected by the Chinese government and was representative of the supply and demand for broiler products at the national level. To measure the COVID-19 Epidemic Severity Index, the number of confirmed Covid-19 cases on the last day of each week was used, sourced from the official website of the National Health Care Commission of the People’s Republic of China. To account for the significant variation in the number of confirmed cases across different periods, the natural logarithm of the COVID-19 severity index (denoted as LnCovid-19) was used to reduce series fluctuations, enhance data linearization, and eliminate the effect of heteroskedasticity.

**Fig 2:**
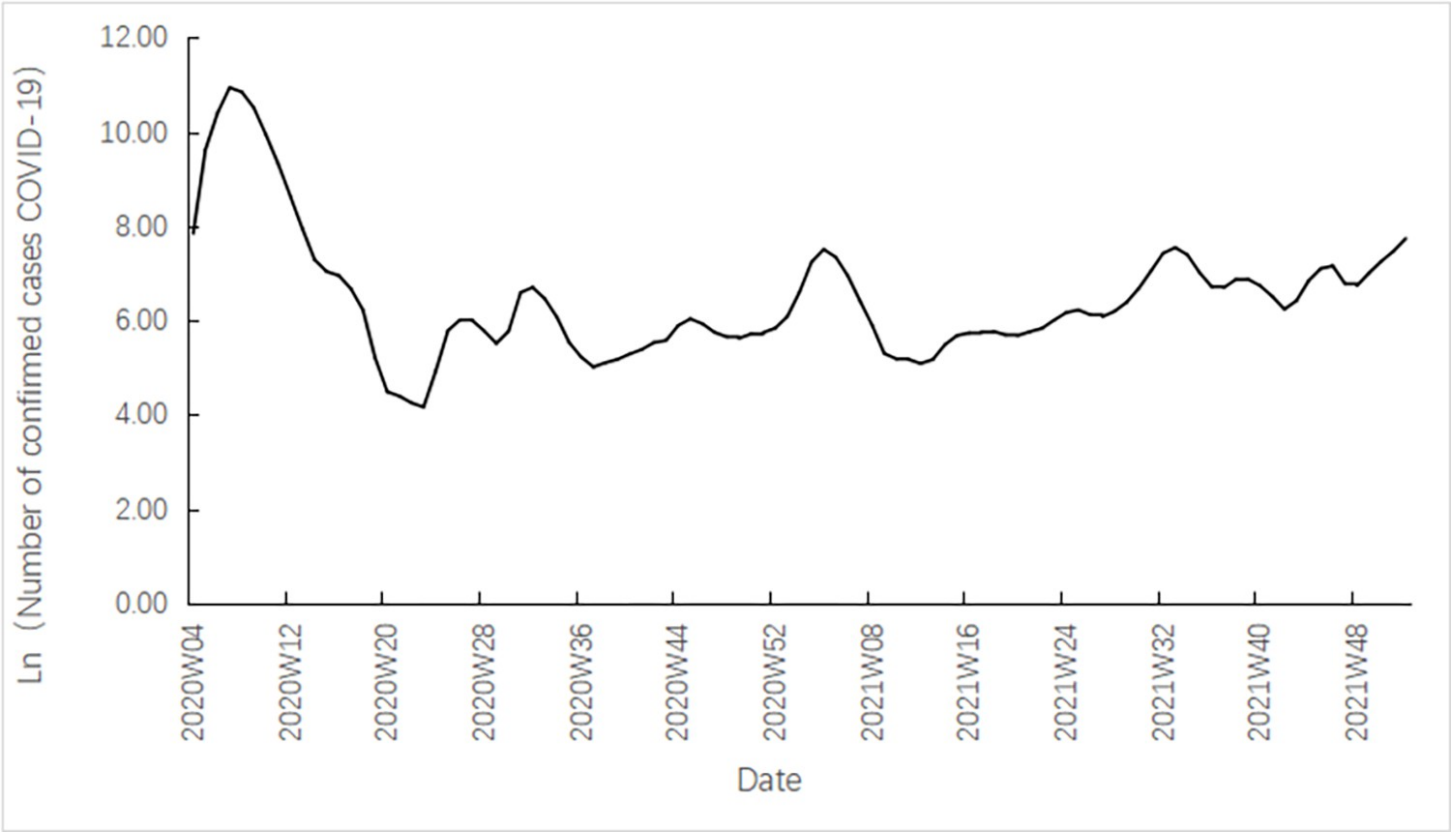
COVID-19 epidemic severity index.

As shown in Fig 2, China experienced four distinct waves of the COVID-19 pandemic between Week 4 of 2020 and Week 52 of 2021. The first wave occurred from January to March 2020 and was the most severe, resulting in over 50,000 new infections in a single day and many fatalities. However, this wave was mostly confined to Hubei Province. Subsequent waves, which took place in August 2020, January 2021, and August 2021, were relatively mild but had a more dispersed spread throughout the country. The COVID-19 severity index developed in this study aligns with the actual situation and can accurately reflect the severity of the pandemic, making it a valuable tool for examining the impact of the COVID-19 shock on price volatility in the broiler industry chain in China.

**Fig 3:**
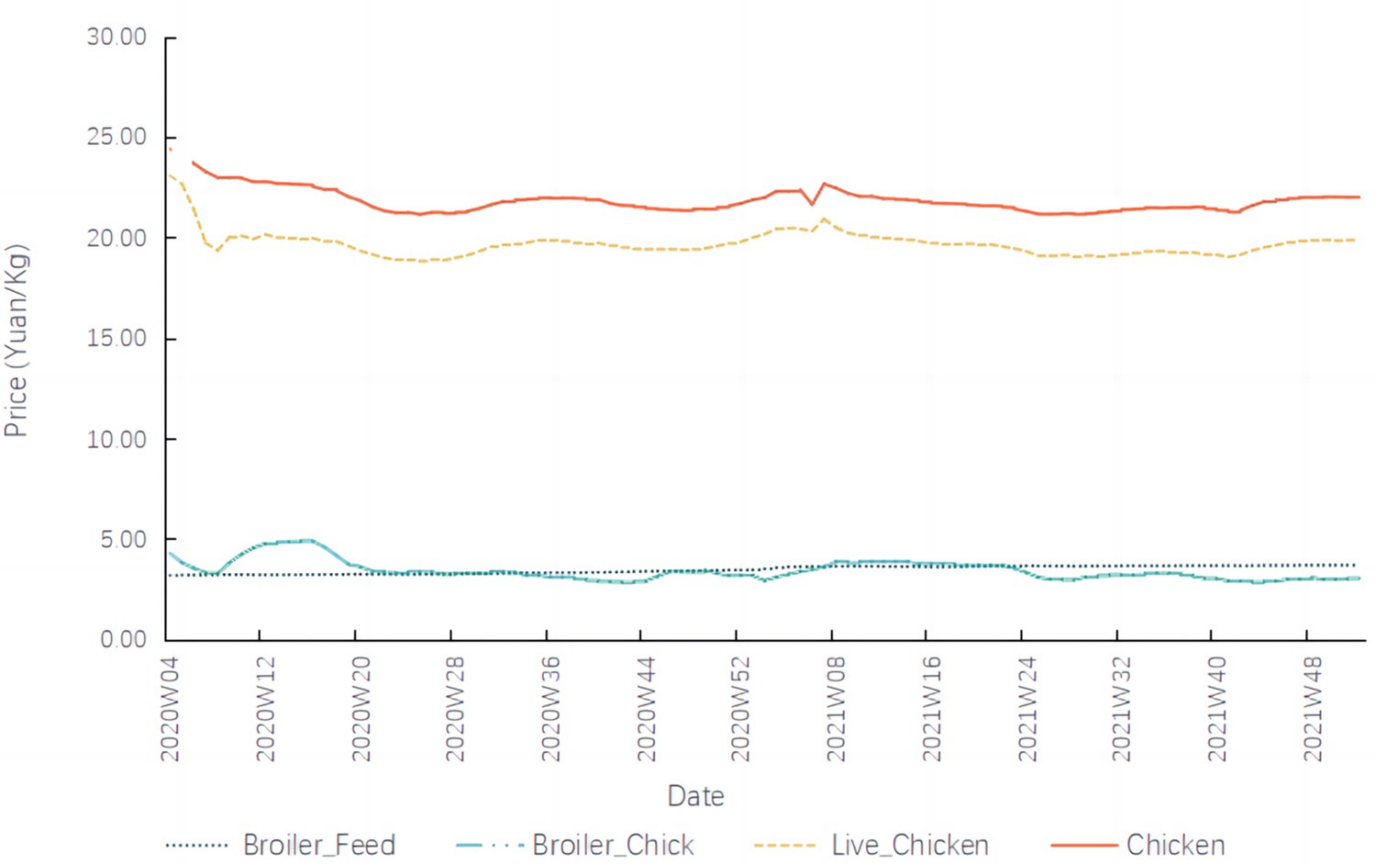
Broiler product prices.

As illustrated in Fig 3, the prices of China’s broiler industry chain maintained relatively stable overall since the COVID-19 outbreak. Nevertheless, temporary fluctuations have been observed. Among these, broiler feed prices exhibited the mildest fluctuations, while the frequency and intensity of fluctuations in prices for broiler chicks, live chickens, and chicken were considerably higher than those for broiler feed.

### Econometric methods

Given the varying impacts of the COVID-19 pandemic and shifting broiler market prices, sudden structural changes may arise. Referring to the study of Kang et al. [44], we utilized a time-varying parameter vector autoregressive (TVP-VAR) model for empirical analysis. The TVP-VAR model was initially proposed by Primiceri [45] and later improved by Nakajima [46] in 2011. Compared to traditional linear or fixed coefficient models that often struggle with estimation performance and are susceptible to systematic errors, the TVP-VAR model depicts dynamic relationships among variables by employing time-varying parameters. Doing so effectively characterizes and illustrates fluctuations in different variables, producing more accurate presentations of the changes over time [37].

The TVP-VAR model utilized to examine the COVID-19 epidemic and price fluctuations in broiler products is established as follows [47, 48].


$$y_{t}=c_{t}+B_{1t}y_{t-1}+\cdots +B_{st}y_{t-s}+e_{t},\;e_{t}∼N(0,\Omega_{t})$$ (3)

In Eq (3), t denotes time and s denotes the number of lags, where t = s+1, s+2,⋯,s+n. The observable endogenous vector is denoted by y_t_ and the vector of time-varying constant terms is denoted by c_t_. Both y_t_ and c_t_ are k×1 dimensional. The matrix of time-varying coefficients, B_1t_,…, B_st_, is k×k dimensional. Meanwhile, the matrix of time-varying covariances denoted by Ω_t_, where $\Omega_{t}=A_{t}^{-1}\sum_{t}\sum_{t}^{,}A_{t}^{,-1}$, is also k×k dimensional. The lower triangular matrix A_t_ with diagonal 1 is defined as follows.


$$A_{t}=\left[\begin{array}{cccc} 1 & 0 & \cdots & 0\\ a_{21} & 1 & 0 & 0\\ \vdots & 0 & \ddots & \vdots \\ a_{n1} & a_{n2} & \cdots & 1 \end{array}\right]$$ (4)

The matrix ∑_*t*_ is symmetric and can be represented as ∑_t_ = diag(σ_1t_,⋯,σ_kt_). By defining the vector βt as the set of B1t, …, Bst, Eq (3) can be transformed into the following form.


$$y_{t}=X_{t}^{,}\beta_{t}+A_{t}^{-1}\sum_{t}\varepsilon_{t}$$ (5)

In Eq (5), $X_{t}^{,}=I_{n}⊗[1,y_{t-1}^{,},\cdots ,y_{t-k}^{,}]$, where ⊗ is the Kronecker product. *h*_t_ = (*h*_1t_,⋯*h*_kt_), where $h_{it}=\log\sigma_{it}^{2}$. The covariance matrices for the time-varying parameter perturbation terms Σβ, Σa, and Σh are all diagonal matrices. The time-varying parameters follow a random wandering process as described below.


$$\left[\begin{array}{c} \beta_{t+1}=\beta_{t}+\mu_{\beta t}\\ \alpha_{t+1}=\alpha_{t}+\mu_{\alpha t}\\ h_{t+1}=h_{t}+\mu_{ht} \end{array}\right]\left[\begin{array}{c} \varepsilon_{t}\\ \mu_{\beta t}\\ \mu_{\alpha t}\\ \mu_{ht} \end{array}\right]∼N\left[0,[\begin{array}{cccc} 1 & 0 & \cdots & 0\\ 0 & \Sigma_{\beta } & 0 & 0\\ \vdots & 0 & \Sigma_{\alpha } & \vdots \\ 0 & 0 & \cdots & \Sigma_{h} \end{array}]\right]$$ (6)

## Results and discussion

### Stability test

To avoid the pseudo-regression phenomenon, it is essential to ensure the validity of the data prior to fitting the dynamic regression model. This study conducted an ADF unit root test on the data sets mentioned above to verify the stationarity of the time series, and the results are presented in Table 1. Although the ADF values of the original data for Broiler-feed and Chicken variables were non-stationary time series and exceeded the critical values at the 5% significance level, they became stationary at the 5% significance level after being subjected to first-order difference treatment, indicating the absence of unit root phenomenon. Hence, the variables could be utilized in developing the TVP-VAR model, and the estimation validity could be ensured.

**Table 1:** ADF unit root test results.

Variable	ADF statistic	(C,T,K)	5% threshold value	Conclusion
Broiler-Feed	-1.609417	(C,T,1)	-3.455842	Unstable
Broiler-Chick	-3.714905	(C,T,2)	-3.456319	Stable
Live-Chicken	-3.983293	(C,0,2)	-2.891234	Stable
Chicken	-3.907196	(C,T,0)	-3.455376	Unstable
LnCovid-19	-3.867666	(C,T,1)	-3.455842	Stable
D(Broiler-Feed)	-5.496511	(C,0,0)	-2.890926	Stable
D(Broiler-Chick)	-5.616665	(0,0,0)	-1.944105	Stable
D(Live-Chicken)	-5.806965	(0,0,0)	-1.944105	Stable
D(Chicken)	-10.69258	(C,T,0)	-3.455842	Stable
D(LnCovid-19)	-5.999071	(0,0,0)	-1.944105	Stable

Note: In Table1, C represents the constant term, T represents the trend term, K represents the lag order, and D() represents the first-order difference of each sequence variable.

### Optimal lag order determination

This study employed various methods to ascertain the optimal lag order of the model, ingcluding the log likelihood (LogL), likelihood ratio statistic (LR), final prediction error (FPE), chi-square information criterion (AIC), Schwartz criterion (SC), and Hannan-Quinn information quantity criterion (HQ). The optimal lag order is represented by the highest number of asterisks ("*"). As shown in Table 2, the optimal lag order for this model is 3.

**Table 2:** Determination of the model lag.

Lag Period	LogL	LR	FPE	AIC	SC	HQ
0	-183.5729	NA	3.36e-05	3.888101	4.020818	3.941765
1	533.6507	1345.718	2.13e-11	-10.38455	-9.588248	-10.06257
2	591.6875	102.9105	1.08e-11	-11.06572	-9.605833*	-10.47541
3	632.7679	68.60855*	7.84e-12*	-11.39728*	-9.273803	-10.53865*
4	648.3930	24.48473	9.70e-12	-11.20398	-8.416922	-10.07703

Note: "*" indicates the optimal lag order according to corresponding criterions.

### Results of MCMC estimation

In our research, we relied on Nakajima’s study to obtain a valid sample for the TVP-VAR model [46]. We drew 10,000 simulated samples using the Markov chain Monte Carlo (MCMC) method and removed the initial 1000 samples. If all mean values resided within the 95% confidence intervals, and the Geweke convergence diagnostic values fell within the 5% critical value range, it indicates that the parameters have successfully converged towards the posterior distribution. The results of the MCMC estimation in our study (Table 3) showed that the posterior means of all parameter tests were within the 95% confidence interval. Additionally, the Geweke convergence diagnostic value was significantly lower than the critical value of 1.96 at a 5% significance level. The maximum null factor of broiler product price was 83.47, which was much lower than the sampling number and sufficient for posterior inference. These findings suggest that our MCMC sampling was relatively concentrated and that the model fit was satisfactory.

**Table 3:** MCMC parameter estimation results.

Parameters	Mean	SD	95% confidence interval	95% confidence interval	Geweke	Inef.
(Σ_β_)_1_	0.0023	0.0003	0.0018	0.0028	0.043	5.19
(Σ_β_)_2_	0.0023	0.0003	0.0018	0.0029	0.248	6.06
(Σ_α_)_1_	0.0070	0.0021	0.0041	0.0125	0.000	69.95
(Σ_h_)_1_	0.0059	0.0030	0.0034	0.0111	0.135	83.47
(Σ_h_)_2_	0.0056	0.0016	0.0034	0.0095	0.518	20.85

### Equidistant impulse responses

**Fig 4:**
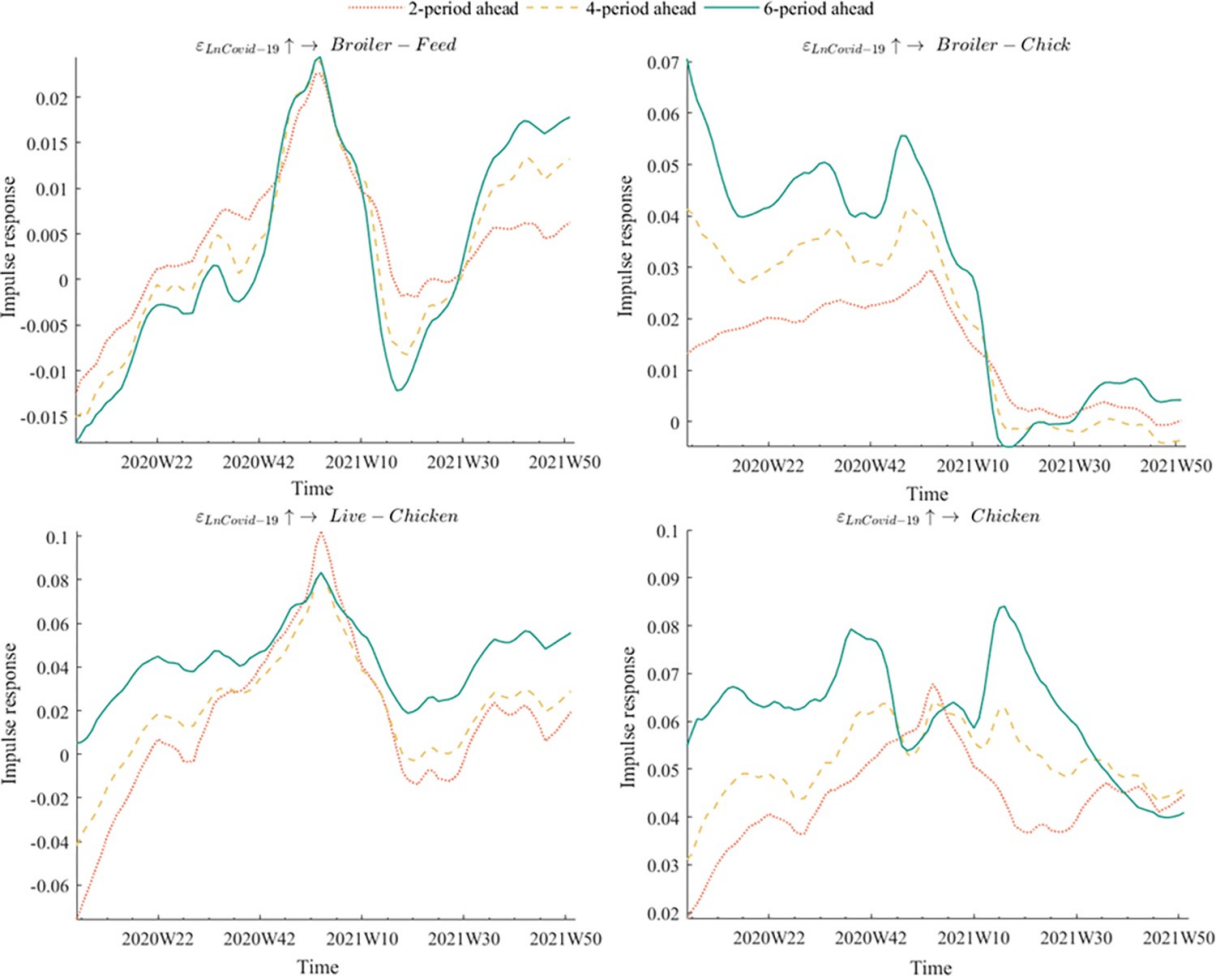
Equidistant impulse responses of COVID-19 epidemic to different broiler product prices.

The time intervals for impulse responses were set at three equidistant levels: 2 periods ahead, 4 periods ahead, and 6 periods ahead. Fig 4 depicted the equidistant impulse response of various broiler product prices to the outbreak of COVID-19 epidemic. The findings demonstrated that live chicken and chicken prices were the most severely impacted, with broiler chick prices following closely behind. In contrast, broiler feed prices experienced relatively minor effects. Two probable reasons explain this trend. Firstly, the original outbreak coincided with the Spring Festival, which happened to be the off-season for feed processing plants. Consequently, poultry farmers and feed processing plants had already stored enough feed to handle short-term supply shortages that could arise from subsequent rounds of the epidemic. Our empirical findings showed that COVID-19 significantly impacted broiler price volatility. This aligns with the observations of Reardon et al. [49] on the broader food supply chain disruptions caused by the pandemic. Secondly, feed is a vital product that benefits from special support policies, such as the green channel. Since the outbreak of the COVID-19 epidemic, the China Grain Reserves Corporation had effectively utilized its extensive vertical management system to ensure efficient coordination, thereby guaranteeing the supply of feed. The introduction of policy-based feed grains had also effectively eased the pressure of grain shortage for breeding enterprises and stabilized broiler feed prices. This aligns with the findings of Gomera et al. [50], which emphasizes the importance of government intervention in stabilizing agricultural inputs during crises.

In terms of impulse response direction, the prices of both broiler chicks and chicken exhibited a positive impulse response regardless of the time lag. On the other hand, there was a "reversal effect" in the impulse response of live chicken prices in response to the COVID-19 epidemic, with a negative trend followed by a positive trend. In the early phase of the epidemic, the negative shock was due to excessive supply and low demand, as transportation and marketing disruptions resulted in overcapacity. Moreover, consumers were hesitant to purchase live poultry products due to safety concerns, while bans on dine-in and restrictions on public gatherings further compounded the decline in demand. However, the medium and long-term trend became positive as broiler producers reduced production and some small and medium-sized producers even exited the market. Furthermore, when the epidemic was brought under control and the restaurant industry resumed normal operations, customer demand for poultry products increased as chicken is rich in nutrients that improve immunity, including protein, lipid-soluble minerals, and vitamins. Carter et al. [51] highlighted the role of policy interventions in supporting market adjustments. The reduction in production and market exits could be viewed as a mechanism of market correction, where policy support and market dynamics interacted to restore equilibrium.

In terms of the impulse responses of different lags, it was observed that the response of broiler product prices to the COVID-19 outbreak was the weakest at lag 2, gradually increasing at lag 4, and becoming the strongest at lag 6. This indicated that the impact of the COVID-19 outbreak on broiler product prices continued to intensify as the lag periods increased. By comparison, the effects of avian flu and African swine fever epidemics on broiler product prices weakened rapidly with increasing lag periods as they only affect the poultry industry. Unlike these two diseases, the COVID-19 was a public health emergency with a more comprehensive impact on society, including on feed, human movement, and other factors. Consequently, it created a "cumulative effect" where the impact became more significant with a more extended period.

### Point-in-time impulse responses

**Fig 5:**
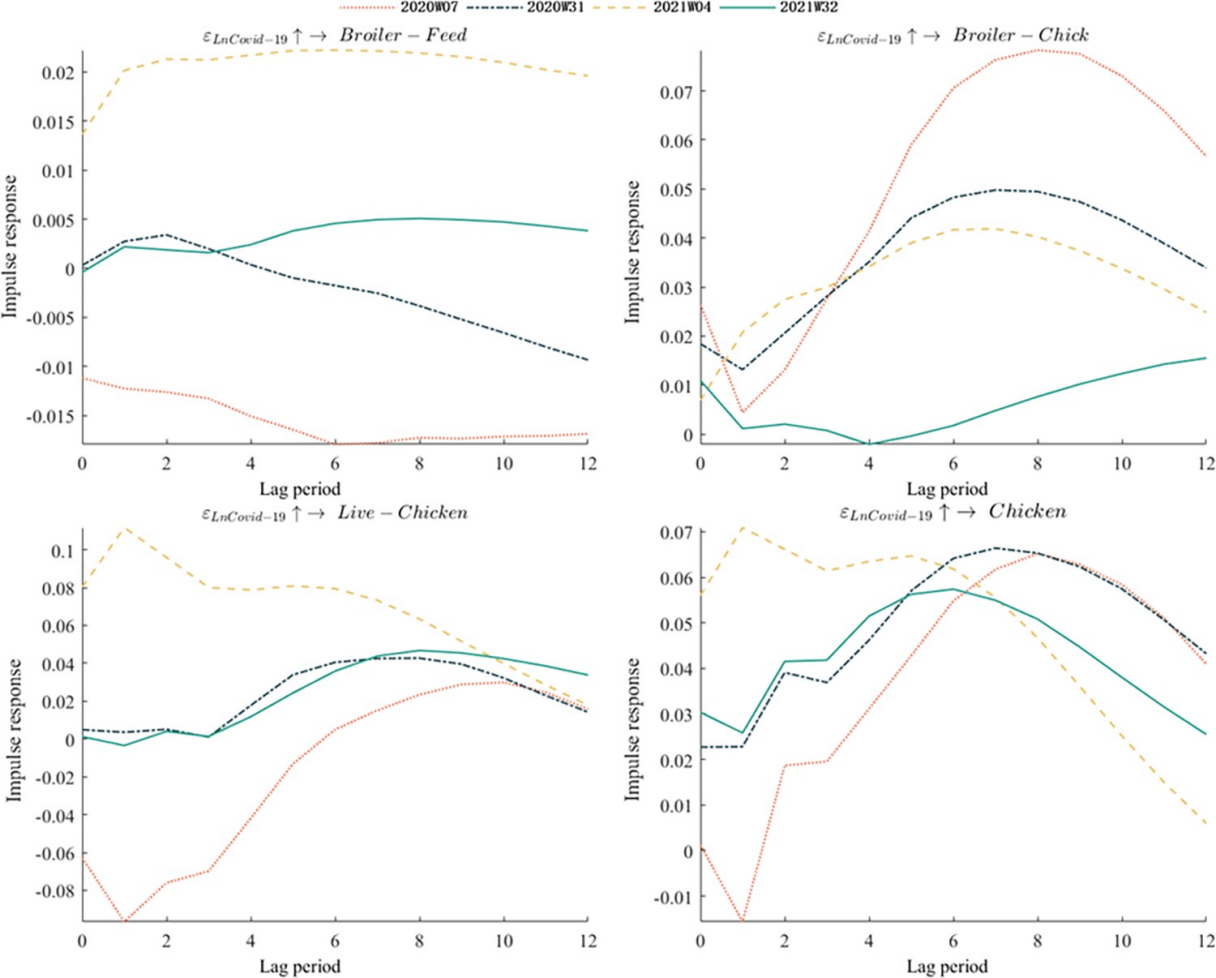
Time point impulse responses of COVID-19 epidemic to different broiler product prices.

Fig 5 presented the point-in-time impulse responses of various broiler product prices to the COVID-19 epidemic outbreak. This study examined four specific time points during throughout the outbreak, namely the 7^th^ week of 2020, the 31^st^ week of 2020, the 4^th^ week of 2021, and the 32^nd^ week of 2021. These time points coincided with the four peak periods of the epidemic. Results indicated that the COVID-19 epidemic had a significant impact on broiler chick prices, live chicken prices, and chicken prices, but only a minor effect on broiler feed prices, aligning with the prior analysis. The direction of impulse responses varied at different time points during the epidemic. Since the COVID-19 epidemic had a negligible impact on broiler feed prices, this paper did not discuss its direction. Positive impulse responses were observed in broiler chick prices and chicken prices during all four time points, while live chicken prices exhibited predominantly negative impulse responses during the 7th week of 2020, but positive impulse responses during the other three time points. This difference in timing could be attributed to the varying outbreak prevention and control policies implemented by the government at different stages. During the first wave, the government imposed strict lockdown measures to contain the outbreak, causing a sharp decline in live chicken production capacity due to plant shutdowns and bans on live poultry trading. However, subsequent waves were met with a series of support policies based on previous experiences, such as resuming broiler factories and normalizing restaurant operations, facilitating a quick rebound in broiler consumption demand. Despite the increased demand, broiler supply was limited by production cycles and risk-averse producers, making it challenging to return to normal levels within a short period.

### Robustness test

**Fig 6:**
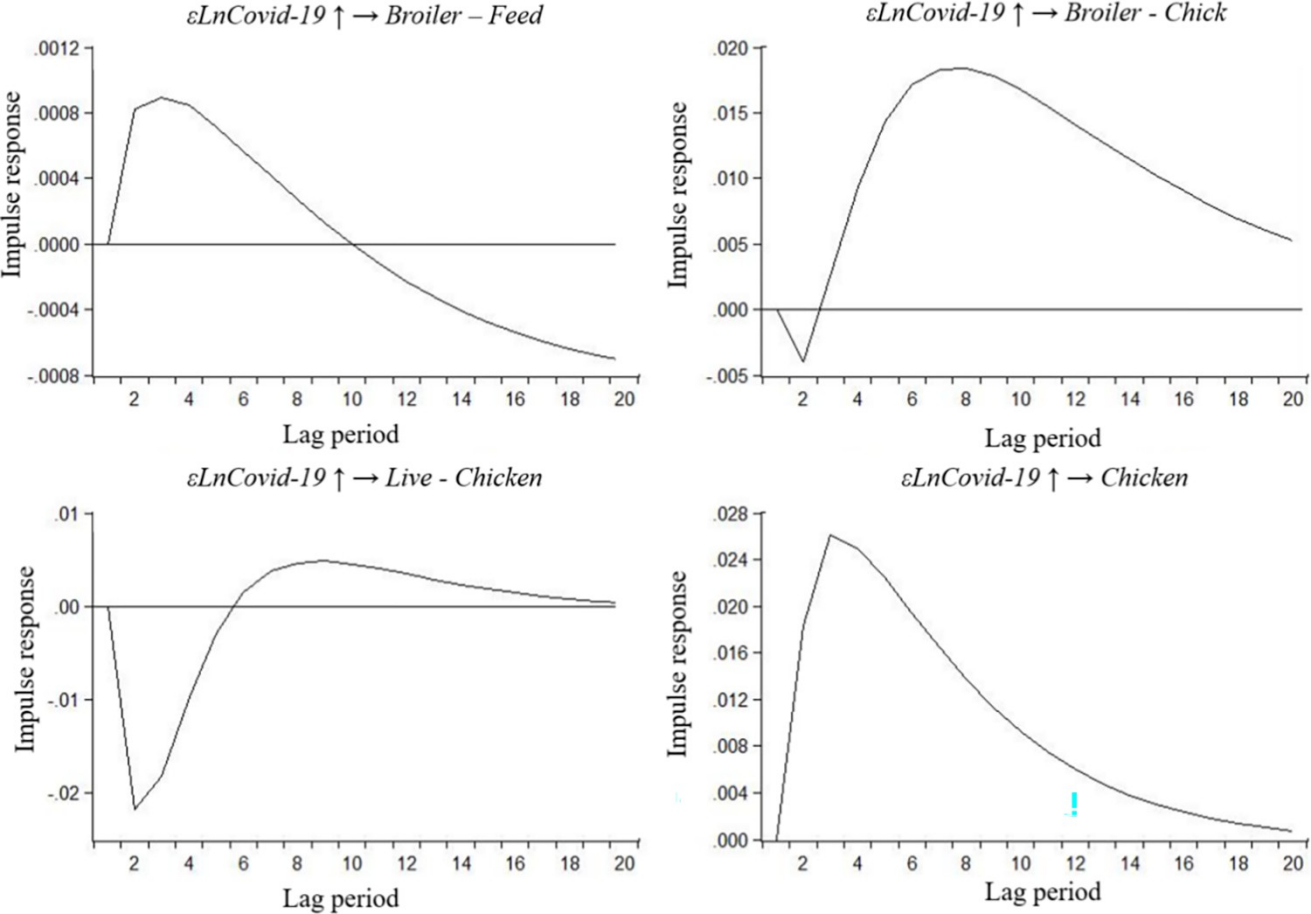
Impulse responses of COVID-19 epidemic to different broiler product prices by BVAR mode.

To further enhance the credibility of our empirical findings, we employed two distinct methods. Firstly, we utilized the Bayes vector auto-regression (BVAR) model to investigate the impact of COVID-19 pandemic on various broiler product prices. As shown in Fig 6, the response results indicate that broiler feed prices exhibited minimal impact by the epidemic. In contrast, broiler chick and chicken prices demonstrated a positive response and ultimately converged to zero. On the other hand, live chicken prices experienced a significant inverse effect during the epidemic, with a negative short-term response transitioning into a positive long-term response. These results provided further evidence in support of our previous empirical research.

**Fig 7:**
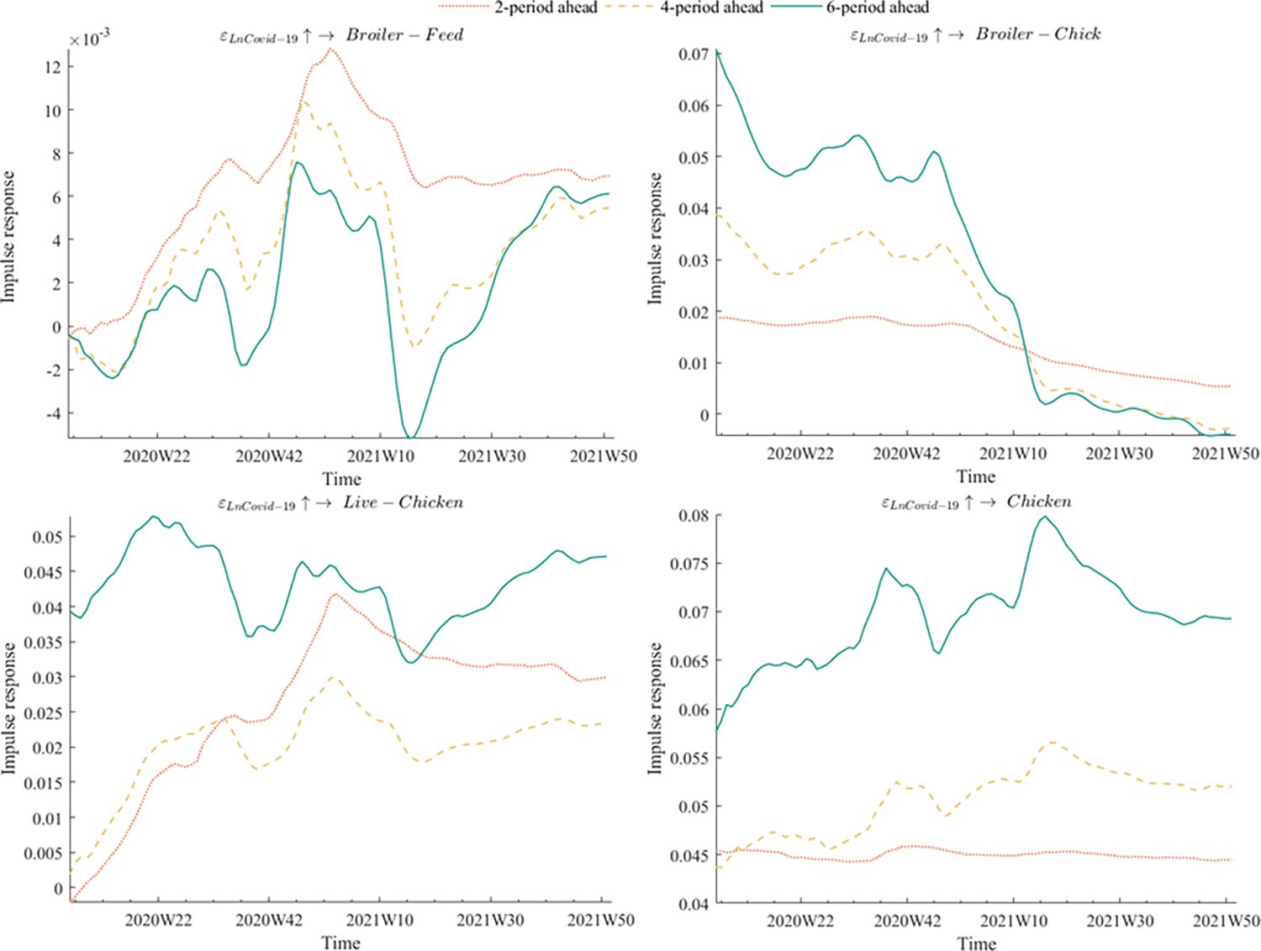
Equidistant impulse responses of COVID-19 epidemic to different broiler product prices by substitute variable.

Secondly, we replaced the variable of confirmed cases with a different substitute variable (average number of daily confirmed cases per week) and consistently utilized the TVP-VAR model to examine the dynamic impulse responses of different broiler product prices to this substitute variable. As evidenced by the results presented in Fig 7, the impulse response outcomes of the substitute variable were consistent with the original variable estimation results, also confirming the robustness of our analysis.

## Conclusions and policy recommendations

### Conclusions

This study employed the number of confirmed COVID-19 cases as a proxy variable to measure the impact of the epidemic on the broiler industry chain in China. Data on weekly prices for each link of the industry chain were collected from January 2020 to December 2021, and a TVP-VAR model was utilized to conduct a thorough analysis. Our findings demonstrated that the impact of the COVID-19 epidemic on the prices of various broiler products varied in different lag periods and time points, both in terms of magnitude and direction. Specifically, live chicken and chicken prices were more affected than broiler feed prices. The impact on broiler chick and chicken prices was predominantly positive, while the impact on live chicken prices had a "reversal effect," with a negative impact in the short term and a positive impact in the medium and long term. The impact of the epidemic on broiler product prices continued to escalate, with the impulse response of lag 6 being greater than lags 4 and 2. The impact on broiler chick and chicken prices remained positive throughout each wave of the COVID-19 outbreak, while the impact on live chicken prices was mostly positive during the same time periods, barring the predominantly adverse effects observed during the initial wave of the COVID-19 outbreak. These discrepancies in impact could be primarily attributable to alterations in supply and demand resulting from evolving epidemic prevention and control policies.

### Policy recommendations

In order to mitigate the impact of unforeseen public events on broiler breeding, stabilize chicken prices, guarantee farmers earnings, and sustain the steady progress of the broiler sector, several measures are proposed in this paper. Firstly, it is recommended to establish a global monitoring mechanism and facilitate the timely dissemination of reports to enhance the monitoring and early warning capabilities of the broiler market. These recommendations align with the findings of Béné et al. [52] and Tendall et al. [53], which emphasized the importance of timely information dissemination and proactive measures in enhancing resilience. Secondly, the promotion of modern breeding, processing, and distribution methods coupled with improved global agricultural trade cooperation and diversified import patterns will strengthen the broiler industry’s ability to cope with uncertainty. These recommendations are consistent with the strategies suggested by Altieri et al. [54] and Laborde et al. [24], highlighting the role of technological advancement and diversified trade patterns in mitigating the adverse impacts of shocks. Thirdly, emergency management systems for public health crises should be developed, including timely publication of situation updates and corresponding response plans. Furthermore, pro-cyclical and counter-cyclical broiler policy support and control systems should be implemented. Lastly, the efficiency and effectiveness of prevention and control plans for public health emergencies should be comprehensively evaluated, and resources should be allocated through a scientific cost-benefit analysis.

### Research limitations and perspectives

Although this study has made significant strides in revealing the dynamic impact of the COVID-19 epidemic on price volatility in the broiler industry chain in China, several shortcomings remain. First, the issue of data timeliness: due to the persistence and complexity of the epidemic’s impact on the market, the data used in this study may not fully capture the long-term dynamics of the epidemic’s effect on the broiler industry chain. Second, the study does not fully account for regional differences. Variations in the spread of the epidemic and the preventive measures taken in different regions may lead to inconsistent market responses, which affects the generalizability of the study’s conclusions.Future research should address these limitations and expand in the following areas. First, enhance the timeliness and coverage of the data to track the evolving long-term impacts of the epidemic on the broiler industry chain, thereby improving the timeliness and accuracy of the findings. Second, increase the analysis of regional differences to study the varying market responses in different regions during the epidemic. This will provide more targeted policy recommendations and a more comprehensive understanding of the broiler industry’s dynamics under the influence of public health crises.

## Supporting information

S1 Data (XLSX)
